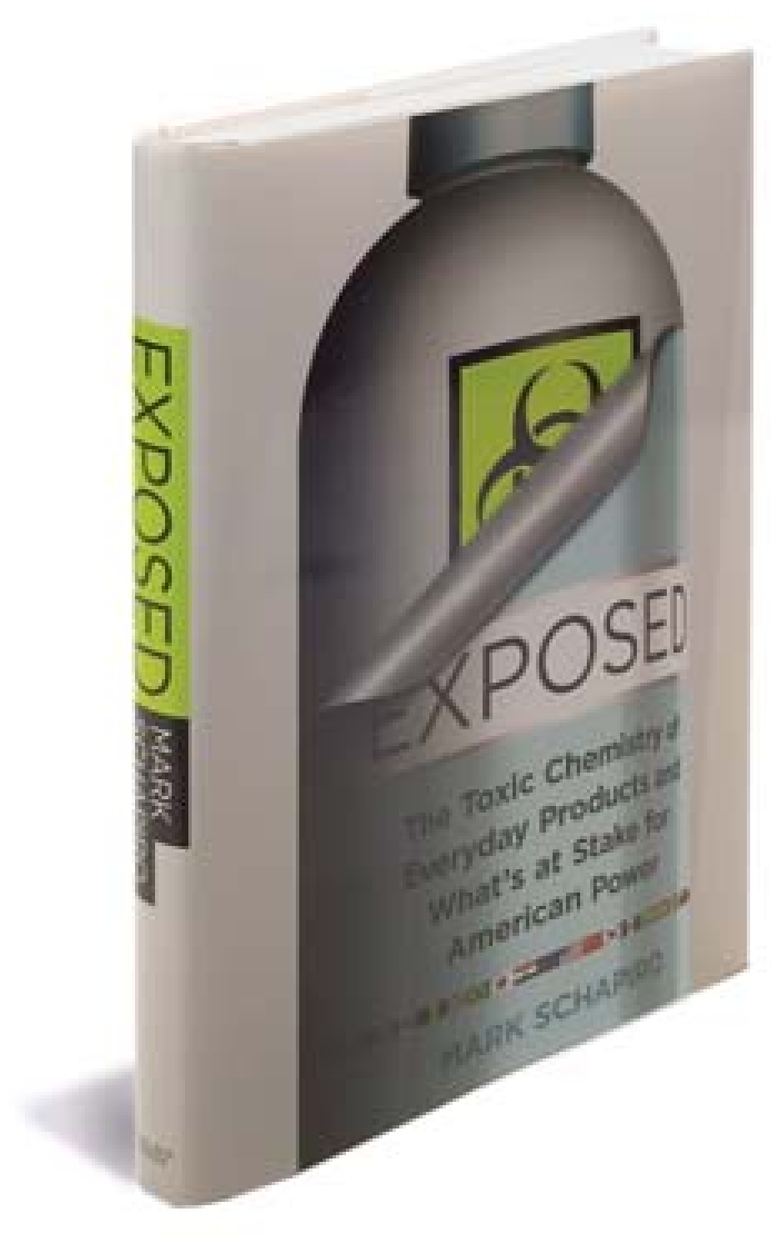# Exposed: The Toxic Chemistry of Everyday Products and What’s at Stake for American Power

**Published:** 2008-05

**Authors:** C.W. Jameson

**Affiliations:** C.W. Jameson is currently an independent consultant on environmental carcinogenesis and public health. He recently retired from the National Institute of Environmental Health Sciences, where he was the Director of the National Toxicology Program’s Report on Carcinogens

*Exposed* is a book with a long subtitle that provides nontechnical information on a topical and current issue. The book, written for the lay public, recounts the recent failures of the United States in protecting the environment and public health and how the country has lost its leadership role in these areas to Europe; thus, Americans are being exposed to environmental hazards to which their European counterparts are not.

Schapiro’s method is to assess recent decisions made by U.S. regulators and policy makers that have had major environmental and economic impacts in the United States. The author describes actions taken on specific toxicants such as mercury, cadmium, lead, chromium, poly-chlorinated biphenyls, and phthalates that have been banned by the European Union (EU) but are still allowed, at least to some extent, by the United States. In another example, the lack of consensus about the safety of genetically modified organisms has caused a significant economic impact in the United States.

Schapiro provides an interesting account of how the EU expanded to 27 countries in 2007 and created a unified market that is now larger than the United States. He describes the coalescing of Europe into a powerful economic and political bloc with environmental health priorities in its policy making, and the simultaneous retreat from such policies in the United States. This eye-opening account demonstrates how the EU became the global environmental leader and why it can now dictate that manufacturers adapt to Europe’s standards to gain market access. U.S. multinational companies have had to adjust as the United States continues to expose its citizens both to environmental hazards and to economic consequences while other global powers such as China follow the EU lead.

Schapiro’s experience in addressing environmental concerns is evident in his approach. For example, he uses the fact that a number of reported carcinogens, mutagens, and neurotoxins have been banned from cosmetics in Europe but are still permitted in cosmetics in the United States to show that officials in the different locations work with the same toxicology data and the same scientists, but reach entirely different conclusions on whether to act on dangerous chemicals. In another chapter, Schapiro shows how phthalates have been identified as toxic (potential human developmental toxicants) yet are still permitted in American toys while banned in Europe. The irony that Americans are now benefiting from the protections of European, not American, laws as the electronics industry reformulates its production practices according to regulations emanating from the EU and not the United States is very telling. The concluding sentence of the book—“The United States is no longer where it likes to imagine itself to be, at the center of a universe around which the rest of the world revolves”—sums up what has been documented in this book and points to one of the main issues currently facing the United States.

The book’s title was initially misleading: I expected, as a chemist, to read about the chemistry of consumer products as it related to their potential toxicity. However, I quickly saw the book’s focus on the shift in global economic and environmental influence from the United States to Europe and the fact that the American public is generally unaware that this has happened. I found myself becoming more interested in the geopolitical and economic issues surrounding the protection of our environment. This is an aspect of the “environmental” issue that scientists often overlook but should consider, because it is their research and data that form the basis for the regulatory decisions that affect the economics and politics of the world. The book is well written and appears to be largely accurate. (One mistake, however, is on page 44; in fact, Dr. Earl Gray’s research on phthalates was conducted for the U.S. Environmental Protection Agency, published in the peer-reviewed literature, and then provided to the National Toxicology Program’s Center for the Evaluation of Risks to Human Reproduction).

This is a good and very readable book on the position the EU has taken in protecting the health of its citizens—and how the United States must now play catch-up. This book would be a good addition to anyone’s library. It provides a good resource for information on how environmental concerns can affect economics around the world and how the United States has fallen behind as a world leader in environmental health issues.

## Figures and Tables

**Figure:**